# Genetic landscape of ESBL producing international clone ST410 of *Escherichia coli* from pediatric infections in Shenzhen, China

**DOI:** 10.3389/fcimb.2024.1403234

**Published:** 2024-09-11

**Authors:** Sandip Patil, Liu Pai, Hongyu Chen, Yunsheng Chen, Li Xinye, Shaowei Dong, Sanket Kaushik, Bruno Silvester Lopes, Xiaowen Chen, Sixi Liu, Feiqiu Wen

**Affiliations:** ^1^ Department of Haematology and Oncology, Shenzhen Children’s Hospital, Shenzhen, China; ^2^ Paediatric Research Institute, Shenzhen Children’s Hospital, Shenzhen, China; ^3^ Department of Laboratory Medicine, Shenzhen Children’s Hospital, Shenzhen, China; ^4^ Department of Paediatric, The People’s Hospital of Guangxi Zhuang Autonomous Region, Nanning, China; ^5^ Department of Biotechnology, Amity University, Jaipur, India; ^6^ School of Health and Life Sciences, Teesside University, Middlesbrough, United Kingdom; ^7^ National Horizons Centre, Teesside University, Darlington, United Kingdom

**Keywords:** pediatric patients, antimicrobial susceptibility, WGS, cephalosporin resistance, international high-risk clone ST410, *E. coli*, ESBL

## Abstract

**Background:**

The emergence of ESBLs producing cephalosporin-resistant *Escherichia coli* isolates poses a threat to public health. This study aims to decipher the genetic landscape and gain insights into ESBL-producing *E. coli* strains belonging to the high-risk clone ST410 from pediatric patients.

**Methods:**

29 *E. coli* ST410 isolates were collected from young children and subjected to antimicrobial susceptibility testing, Whole-genome sequencing (WGS), serotype analysis, MLST, ESBL genes, virulence genes, and plasmid profiling.

**Results:**

Antimicrobial susceptibility testing demonstrated a high level of resistance to cephalosporins followed by aminoglycoside, sulfonamide, carbapenem and penicillin group of antibiotics. However, n=20/29 shows MDR phenotype. Phylogenetic group B2 (n=15) dominated, followed by group D (n=7), group A (n=4), and group B1 (n=3). Serotyping analysis identified O1:H7 (n=8), O2:H1 (n=6), O8:H4 (n=5), O16:H5 (n=4), and O25:H4 (n=3). Other serotypes identified included O6:H1, O15:H5, and O18:H7 (n=1 each). The most commonly detected ESBL genes were *bla*_CTX-M_, (n=26), followed by *bla*_TEM_ (n=23), and *bla*_SHV_ (n=18). Additionally, *bla*_OXA-1_ (n=10), *bla*_OXA-48_ (n=5), *bla*_KPC-2_ (n=3), *bla*_KPC-3_ (n=2), *bla*_NDM-1_ (n=4), *bla*_NDM-5_ (n=1), *bla*_GES-1_ (n=2), *bla*_GES-5_ (n=1), and *bla*_CYM-1_ (n=3). Notable virulence genes identified within the ST410 isolates included *fimH* (n=29), *papC* (n=24), *hlyA* (n=22), and *cnf1* (n=18), among others. Diverse plasmids were observed including IncFIS, IncX4, IncFIA, IncCol, IncI2 and IncFIC with transmission frequency ranges from 1.3X10^-2^ to 2.7X10^-3^.

**Conclusion:**

The ST410 clone exhibited a complex resistance profile, diverse serotypes, the presence of specific resistance genes (ESBL genes), virulence gene repertoire, and diverse plasmids. The *bla*_CTX-M_ was the most prevalent ESBL gene detected.

## Introduction

The emergence and widespread dissemination of extended-spectrum β-lactamases (ESBLs) in *Escherichia coli* (*E.* coli) pose a significant global public health threat (Antimicrobial Resistance Collaborators, 2022). These enzymes confer resistance to a wide range of β-lactam antibiotics, including cephalosporins, thereby compromising their effectiveness in clinical settings (Bush and Bradford, 2020). Of particular concern is the rapid spread of ESBL-producing strains belonging to high-risk clones, such as *E. coli* sequence type 410 (ST410), which have been associated with multidrug resistance phenotypes (Mazumder et al., 2021). Understanding the genetic landscape and resistance patterns of these strains is crucial for effective surveillance, prevention, and treatment strategies. Our previous studies have reported on the prevalence and characteristics of ESBL-producing *E. coli* strains, highlighting the growing concern over their impact on public health specifically the pediatric population. Our previous findings revealed a significant increase in the proportion of ESBL-producing strains over the period, emphasizing the urgent need for targeted interventions to control their spread (Patil et al., 2023c; Patil et al., 2019b). Furthermore, studies focusing on high-risk clones, such as ST410, have provided valuable insights into their prevalence and associated resistance mechanisms. Smith et al., conducted a study that reported a high prevalence of ESBL genes, including *bla*_CTX-M_ and *bla*_TEM_, among ST410 *E. coli* isolates collected from various clinical sources (Smith et al., 2010). Additionally, a study gives initial suggestions for recent interspecies transmission of a new successful clone of ST410 *E. coli* between wildlife, humans, companion animals and the environment (Schaufler et al., 2016). These findings highlight the importance of understanding the specific resistance mechanisms driving the spread of ESBLs within this clone. While previous research has shed light on the prevalence and resistance patterns of ESBL-producing *E. coli*, there remains a limited understanding of the molecular characteristics and resistance profiles of ST410 isolates obtained from young children. Young children represent a vulnerable population, and their increased susceptibility to infections, coupled with limited treatment options due to antimicrobial resistance (Medernach and Logan, 2018; Patil et al., 2023b), necessitates a focused investigation into the dynamics of ESBL-producing ST410 isolates in this age group. ST410 is a high-risk clone that has gained attention due to its association with multidrug resistance and its ability to disseminate ESBL genes (Roer et al., 2018). It has been identified as a globally prevalent clone, implicated in various healthcare-associated infections and community-acquired infections (Mazumder et al., 2021). This strain has been reported in China from the food market and hospital wastewater (He et al., 2023; Ju et al., 2023), best of our knowledge this is the first report from pediatric clinical cases in China. Therefore, this study aims to comprehensively analyze ESBL-producing *E. coli* ST410 isolates obtained from young children, investigating their genetic and phenotypic characteristics, including serotype distribution, ESBL genes, plasmid profiles, and antimicrobial susceptibility patterns. By expanding our understanding of the molecular epidemiology and resistance mechanisms of these strains, we can inform targeted interventions and improve patient outcomes. In this study, WGS allowed us to obtain detailed genomic information, facilitating the determination of phylogenetic relationships, identification of serotypes, and detection of ESBL genes within the 29 *E. coli* ST410 isolates. Serotyping enabled the characterization of the diverse range of serotypes present among the ST410 isolates, providing insights into the genetic heterogeneity within this high-risk clone. This knowledge will enable the development of targeted interventions to mitigate the spread of multidrug-resistant strains and ensure effective treatment outcomes for infected individuals.

## Materials and methods

This study adhered to the principles outlined in the Declaration of Helsinki and was granted approval by the Institutional Ethics Committee (reference number: 2018 [013], dated September 3, 2018) in accordance with international ethical standards. All experimental procedures were conducted in compliance with the biosafety regulations established by the hospital. *Escherichia coli* isolates were obtained from various clinical samples, including urine (n=20), blood (n=3), abdominal secretion (n=3), stool (n=2), and pus (n=1) (**Table 1**). The isolates were collected between June 1, 2021, and June 30, 2022, from the central microbiology laboratory of Shenzhen Children’s Hospital as part of routine hospital investigations. Given the retrospective nature of the study, the Ethics Committee of Shenzhen Children’s Hospital determined that obtaining patients’ or guardians’ consent was not required. To ensure patient confidentiality and comply with the principles of the Declaration of Helsinki, personal identifiers such as names were not used for research purposes. All data were treated as confidential and handled according to ethical guidelines. Therefore, written consent was not obtained.

**Table 1:** Demographic and clinical characteristics ESBL-Producing Cephalosporin-Resistant *Escherichia coli* ST410.

Gender (n=29)		Age Characteristics		Specimen (n=29)		Department (n=29)	
Male	11 (38%)	Mean SD	3 months	Urine	20 (68.96%)	ICU	6 (20.68%)
Female	18 (62%)	Median (IQR)	10.6 years	Pus	5 (17.24%)	Urology & OPD (each)	5 (17.24%) each
		Age rang	1 month to 15 years	Blood	3 (10.34%)	Surgery	3 (10.34%)
				Stool	1 (3.4%)	Nephrology, Orthopedic &Respiratory Medicine	2 (6.89%) each
						Others	4 (13.79%)

SD, standard deviation; IQR, interquartile range; ICU, Intensive care unit; OPD, outpatient department.

### Clinical sampling and identification of isolates

A total of 29 unique isolates were included in this study, with each isolate obtained from a different patient. Relevant demographic information such as gender, age, and hospitalized department was retrieved from the electronic data record room. These strains do not represent all *E. coli* clinical strains isolated during this time but specifically those producing ESBLs with focus on ST410 is due to its clinical significance and contribution to the high ESBL rate observed in our hospital. Specimens were cultured on blood agar and MacConkey’s agar to facilitate bacterial growth. Identification of the isolates was performed using the VITEK^®^2 compact system (BioMerieux, France). Confirmatory identification was carried out using the API-20 test and matrix-assisted laser desorption/ionization time-of-flight mass spectrometry (MALDI-TOF/MS) with the Microflex Biotyper^®^ LT instrument (Bruker Daltonik GmbH, Bremen, Germany) (Patil et al., 2019b). ATCC 29522 *E. coli* was used as the control strain. The isolates were stored in 40% glycerol at -80°C for further studies.

### Antimicrobial testing and detection of ESBLs and MBLs

The antimicrobial susceptibility test (AST) was conducted on all 29 confirmed *E. coli* isolates using the VITEK^®^2 compact system (BioMerieux, France) with the standard AST09 card (software version 9.01). Additionally, the minimum inhibitory concentrations (MICs) for various antimicrobial agents were determined using the broth dilution method. The tested antimicrobial agents included aminoglycosides (amikacin, tobramycin); aminopenicillin (amoxicillin-clavulanate); cephalosporins (2^nd^ generation- cefuroxime, cefoxitin; 3^rd^ generation ceftazidime, ceftriaxone, cefoperazone/sulbactam; 4^th^ generation cefepime; 5^th^ generation ceftolozane/tazobactam); carbapenems (imipenem, ertapenem); fluoroquinolones (levofloxacin); phosphonic group -(Fosfomycin); nitrofurantoin group (nitrofurantoin); sulfonamide group (trimethoprim/sulfamethoxazole); penicillins plus β-lactamase inhibitors (piperacillin-tazobactam, ticarcillin/clavulanate), and glycylcycline (tigecycline). The AST results were accurately interpreted based on the Clinical and Laboratory Standards Institute (CLSI) (M100Ed34 | Performance Standards for Antimicrobial Susceptibility Testing, 34th Edition, n.d.). The multidrug-resistant (MDR) phenotype was classified according to the criteria defined by Magiorakos et al., where MDR was defined as resistance to at least one agent in three or more antimicrobial groups, and extensively drug-resistant (XDR) was defined as resistance to at least one agent in all but two antimicrobial categories (Magiorakos et al., 2012). The production of ESBL was initially determined using the VITEK^®^2 compact system. However, for enhanced accuracy and precision, the results were confirmed using a double-disc synergy test (DDST) and Metallo-beta-lactamase (MBL) production was assessed using the MBL-E-test (IPM-EDTA; AB Biodisk) specifically for ESBL-producing *E. coli* as per our laboratory’s standardized procedure (Patil et al., 2023a). A positive control strain, which had been previously characterized in our laboratory and confirmed to exhibit the desired traits, was employed as a reference for the experiments (Patil et al., 2019b). In addition, *E. coli* ATCC 25922, a well-established negative control strain widely used for comparative purposes, was included in the study.

### Whole-genome sequencing and analysis

Genomic DNA extraction from the *E. coli* isolates was carried out using the QIAamp DNA Mini Kit (Qiagen, Hilden, Germany) following the manufacturer’s instructions. Subsequently, the Nextera XT DNA Library Prep Kit (Illumina, San Diego, CA, USA) was utilized to prepare the whole-genome sequencing libraries. Multiplexed paired-end sequencing was conducted on an Illumina MiSeq instrument using the MiSeq Reagent V3 Kit (2×300 cycles). To assemble the sequence reads, SPAdes 3.9 software on the ARIES Galaxy server (https://w3.iss.it/site/aries/, accessed on April 14, 2023) was employed for *de novo* assembly. MLST analysis was performed based on the *E. coli* MLST website scheme (https://enterobase.warwick.ac.uk/species/index/ecoli, last accessed on April 18, 2023). To analyze *E. coli* ST410 isolated from a children’s hospital globally, we collected data from the Sequence Read Archive (SRA) at NCBI (https://www.ncbi.nlm.nih.gov/sra/) spanning the years 2009 to 2021 (**Supplementary Table 1**). Our analysis was based on reports and databases that identified the sequences as ST410. Our objective was to construct a comprehensive global distribution map for *E. coli* ST410, focusing exclusively on human-origin isolates and excluding isolates from other sources. In silico analysis of the assembled contigs was performed utilizing the available tools at the CGE server. Serotype Finder was employed to predict the serotype of the isolates. Plasmid Finder and ResFinder tools were used to screen the contigs for plasmid and resistance gene content, respectively, at the CGE server. For plasmid subtyping, the replicon allele at the plasmid MLST site was assigned, and the plasmid types were identified using the plasmid MLST database (https://pubmlst.org/plasmid/, accessed on April 21, 2023). Virulence gene identification related to ESBL-*E. coli* was performed using VirulenceFinder at CGE. Phylogenetic group analysis was performed following the Clermont method (Clermont et al., 2013), which utilizes a triplex PCR approach to classify *E. coli* into phylogenetic groups A, B1, B2, and D. The specific genetic markers chuA, yjaA, and the DNA fragment TspE4.C2 were targeted to assign the isolates to the respective groups. This method was chosen due to its accuracy and wide acceptance in the scientific community. The detailed protocol is referenced in Patil et al. (2019).

### Horizontal gene transfer

We conducted an experiment to investigate the transfer of resistance genes through broth mating, focusing on the genetic environment of (ESBLs)-encoding-cephalosporin resistance genes. Plasmid transfer frequency was calculated. We used streptomycin-resistant *E. coli* C_600_ as the recipient strain. Donor and recipient strains were mixed in a 1:1 ratio and incubated at 37°C for 18-24 hours. Transconjugants were selected on Muller–Hinton agar containing 4µg/ml cefotaxime. To confirm the ESBL phenotype in the transconjugants, an E-test was performed followed by PCR assay and sequencing. The primers and protocol were adopted from our lab as outlined in our previous work (Patil et al., 2019a).

## Results

A total of 29 unique isolates were included, Among the isolates, there were (n=18) isolates from female patients and (n=11) isolates from male patients, resulting in a gender distribution. The median age of the patients was 3 months, with an interquartile range (IQR) of 10.66 years, minimum age was 1 month, and the maximum age was 15 years (**Table 1**). *E. coli* isolate colonies appeared round, and medium-sized and exhibited β-hemolysis with ranged from light pink to reddish on the blood agar. The color pink to dark pink, dry and doughnut-shaped colonies on MacConkey agar. The analysis revealed that each of the 29 isolates was positively confirmed through the API 20 E-test (**Supplementary Table 2**). The MALDI-TOF/MS results showed high confidence scores indicating the presence of *E. coli* in all 29 isolates, confirming their identification.

### Antimicrobial susceptibility

**Figure 1:**
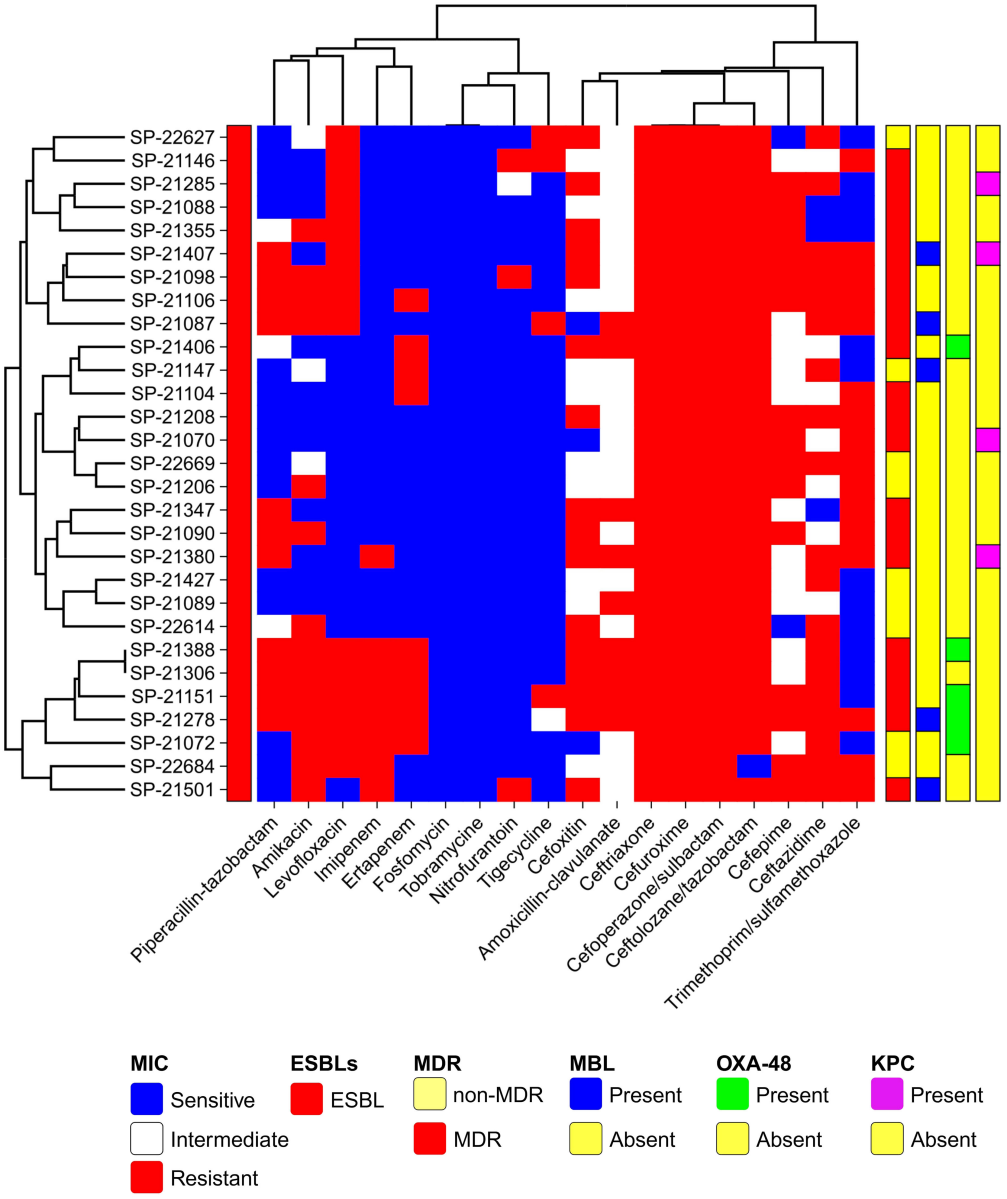
Global Distribution of *E. coli* ST410 Isolates from Human Origin (2009-2021). This map illustrates the reported presence of sequence type 410 (ST410) across different countries. The data represented in this figure are derived from sequence archives and published reports. The data are sourced from external reports and databases. Limitations include potential biases and gaps in the sequence archives, which may affect the accuracy of the global prevalence and distribution depicted.

The antibiotic susceptibility profile of the clinical isolates was evaluated for commonly used antibiotics in clinical practice. The observed resistance patterns among the isolates indicated substantial rates of resistance, particularly to cephalosporins, followed by aminopenicillin, fluoroquinolones, the sulfonamide group, and aminoglycosides. Specifically, resistance rates of 100% (n=29) were noted for 2nd generation cefuroxime, 3rd generation ceftriaxone, cefoperazone/sulbactam, and amoxicillin-clavulanate from the aminopenicillin group. However, 96.55% (n=28) exhibited resistance to 5^th^-generation cephalosporin ceftolozane/tazobactam, followed by 3^rd^-generation ceftazidime at 65.51% (n=19), 2^nd^-generation cephalosporin cefoxitin, and sulfonamide group trimethoprim/sulfamethoxazole at 55.17% (n=16) each. Additionally, 51.72% (n=15) resistance was observed for 4th generation cephalosporin cefepime, aminoglycosides amikacin, and fluoroquinolones levofloxacin each. For penicillins plus β-lactamase inhibitors, piperacillin-tazobactam and ticarcillin/clavulanate exhibited 37.93% resistance (n=11). Carbapenems showed resistance of 31% (n=9) for imipenem and 27.58% (n=8) for ertapenem. Although it is uncommon for *E. coli* to exhibit higher resistance to imipenem than to ertapenem, our data, confirmed through double-checking, indicate this pattern in strains 21380, 22684, and 21501. Further investigation into the resistance mechanisms in these strains may provide insights into this unusual observation (**Figure 1**). Glycylcycline showed a resistance of 20.68% (n=6), while nitrofurantoin exhibited resistance in 10.34% (n=3) of the cases. Notably, the isolates demonstrated 100% sensitivity to fosfomycin and tobramycin (**Figure 1**). Overall, the results indicate widespread resistance among the clinical isolates, particularly against cephalosporins. Out of the total 29 isolates, 68.96% (n=20) exhibited a Multidrug-Resistant (MDR) phenotype, indicating resistance to multiple classes of antibiotics (**Figure 1**). ESBL production was verified in all isolates through the DD-Synergy test. Specifically, 100% of the isolates (n=29) exhibited ESBL production (**Figure 1**). Additionally, among these isolates, further analysis identified 10 isolates as MBL producers, accounting for approximately 34.48% (n=10) of the total isolates tested (**Figure 1**). This highlights a noteworthy subset of isolates within the studied population that possess the MBL phenotype in conjunction with ESBL production.

### Genomic phylogenic analysis

**Figure 2:**
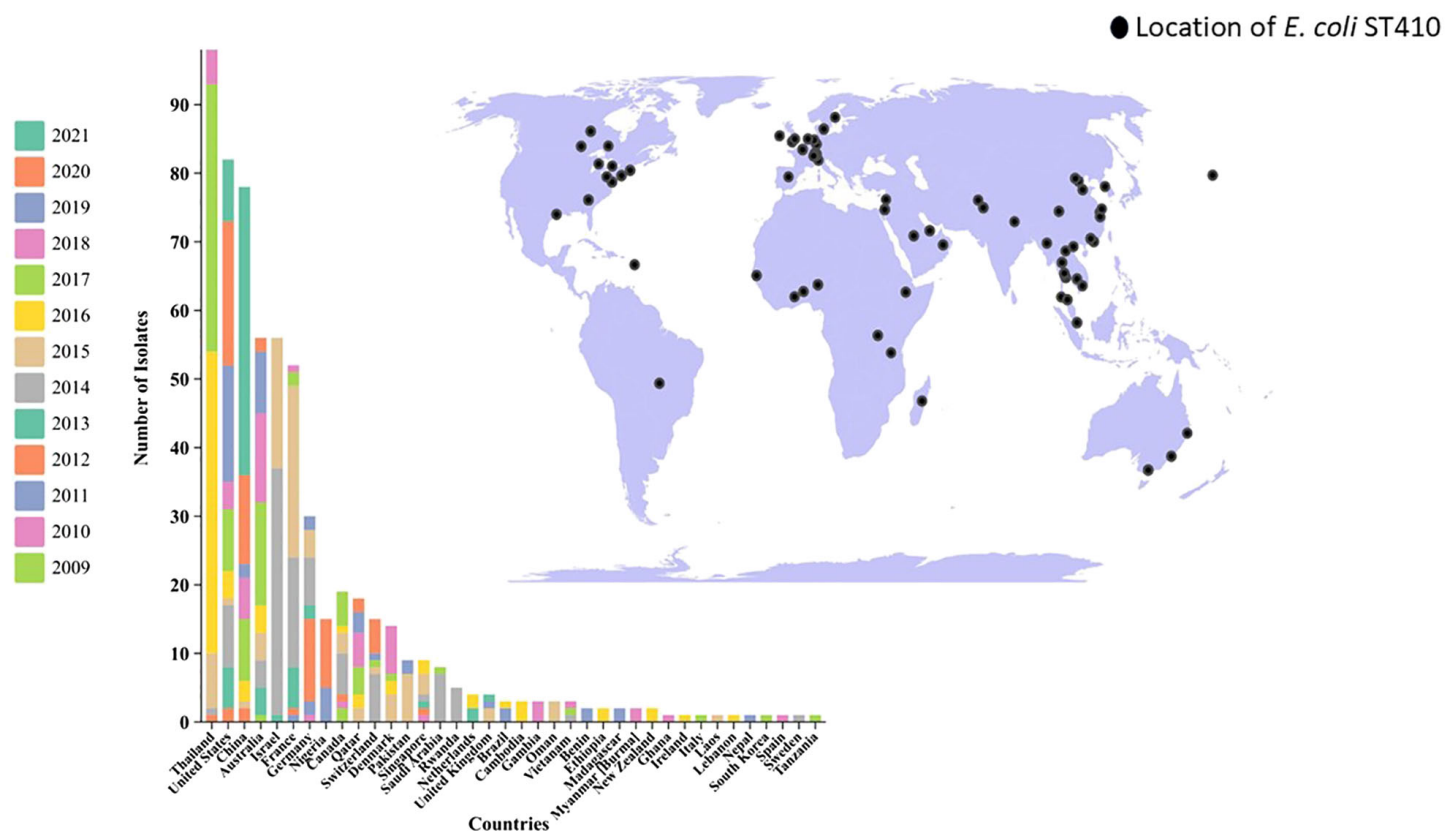
Antibiotic Susceptibility Profile and Phenotypic Characteristics of ESBL-Producing Cephalosporin-Resistant *Escherichia coli* ST410. The heatmap was generated using https://www.chiplot.online/. Last accessed on 15 January 2024.

**Figure 3:**
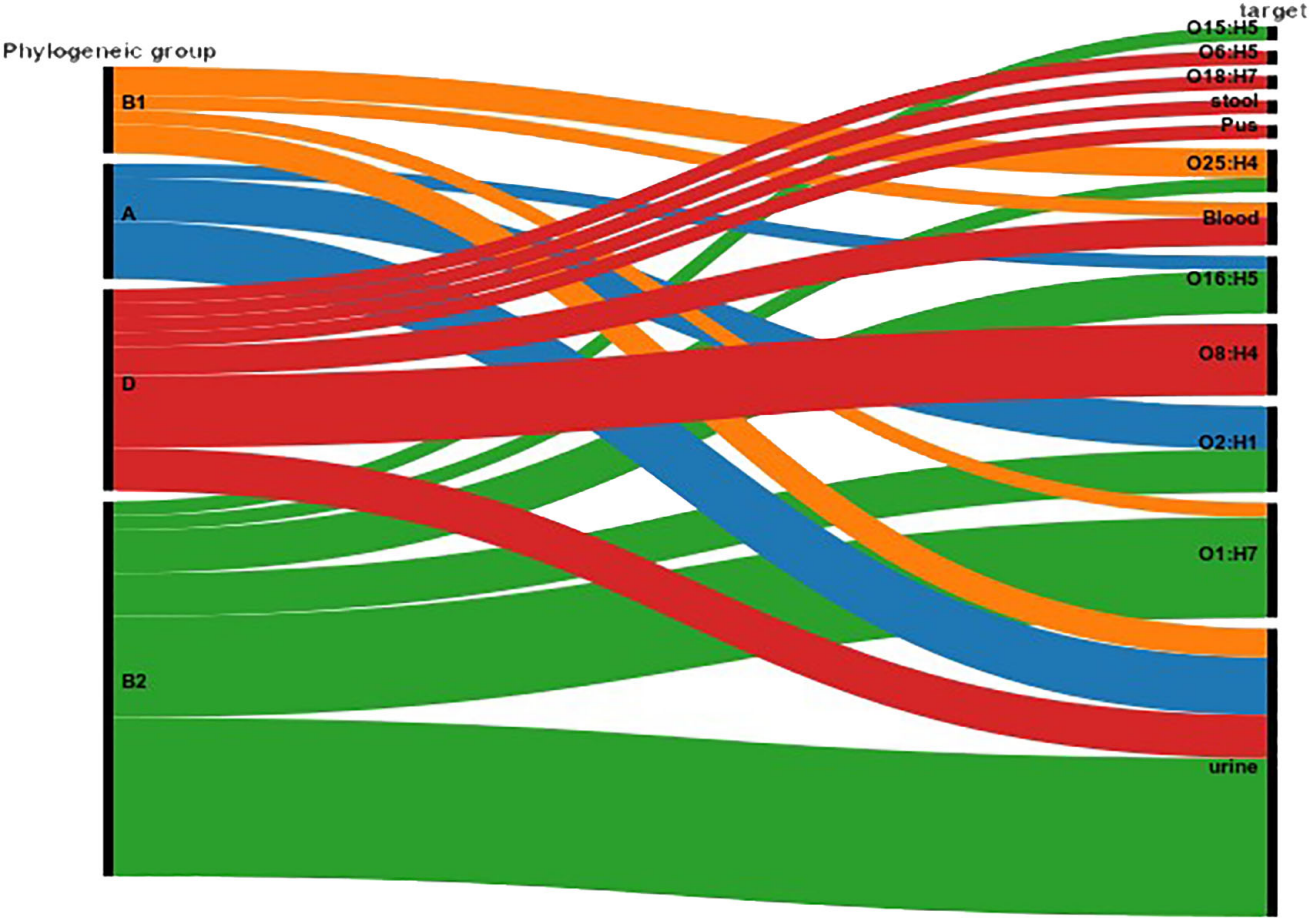
Correlation between Serotype, Phylogenetic Group, and Specimen Source. The figure illustrates the distribution of serotypes across different phylogenetic groups (A, B1, B2, D) and their association with specimen sources (blood, urine, pus, stool). The Sankey Diagram was visualized by RAWGraphs 2.0.1 (https://www.rawgraphs.io), an open-source data visualization framework. Each serotype is represented with its corresponding phylogenetic group, and the distinct specimen origins are indicated by color-coded symbols. This correlation analysis provides insights into the stereotypic diversity and specimen-specific associations within the bacterial population.

MLST analysis based on the *E. coli* MLST website scheme revealed that all 29 isolates in our study belonged to sequence type (ST) 410. This indicates a clonal relationship among the isolates, suggesting a common evolutionary lineage. ST410 is an important sequence type associated with *E. coli* infections. The consistent identification of ST410 among our isolates suggests a potential endemicity or dissemination of this particular sequence type in our study population. Our dataset comprises 607 *E. coli* ST410 isolates, with representation from various regions: with the highest representation observed in Thailand (n=98), followed by the United States (n=82), China (n=78), Australia (n=56), Israel (n=56), France (n=52), Germany (n=30), Niger (n=15), Canada (n=19), Qatar (n=18), Switzerland (n=15), Denmark (n=14), Pakistan (n=9), Singapore (n=9), Saudi Arabia (n=8) and others between 2009-2021. The distribution of isolates from other regions is visualized in **Figure 2**. Phylogenetic group analysis revealed that the majority of the isolates belonged to group B2 51.72% (n=15), indicating a higher association with extraintestinal pathogenic *E. coli* (ExPEC) strains. Group D accounted for 24.14% of the isolates (n=7), followed by Group A at 13.79% (n=4) and Group B1 at 10.34% (n=3). The serotyping analysis identified multiple serotypes among the isolates. The most frequently identified serotype was O1:H7 at 27.59% (n=8), followed by O2:H1 at 20.69% (n=6), O8:H4 at 17.24% (n=5), O16:H5 at 13.79% (n=4), and O25:H4 at 10.34% (n=3). Other serotypes identified by 10.34% included O6:H1 (n=1), O15:H5 (n=1), and O18:H7 (n=1) (**Figure 3**). These findings highlight the distribution and prevalence of phylogenetic groups and serotypes among the isolates in our study population. We established a comprehensive correlation between serotype, phylogenetic group, and specimen sources, as illustrated in **Figure 3**. While we observed a diverse distribution of serotypes within phylogenetic group B2, including O1:H7, O2:H1, O16:H5, and O15:H5. our analysis did not reveal a clear or consistent association between specific serotypes and specimen origins. The D group exhibited a distinct serotype profile, comprising O8:H4, O18:H7, and O6:H5. Remarkably, specimens originating from blood, urine, pus, and stool were associated with this phylogenetic group. Phylogenetic group A demonstrated a specific serotype pattern, with isolates exhibiting O2:H1 and O16:H5, all sourced exclusively from urine specimens. Additionally, isolates in phylogenetic group B1 displayed the O25:H4 serotype, with specimens originating from both blood and urine sources. Despite these observations, no definitive pattern of serotype distribution relative to specimen source was established. These results indicate that while trends in serotype distribution were noted, a significant correlation between specific serotypes and specimen types was not evident. Further investigation may be necessary to better understand the factors influencing serotype distribution and specimen associations.

### Drug resistance genes

**Figure 4:**
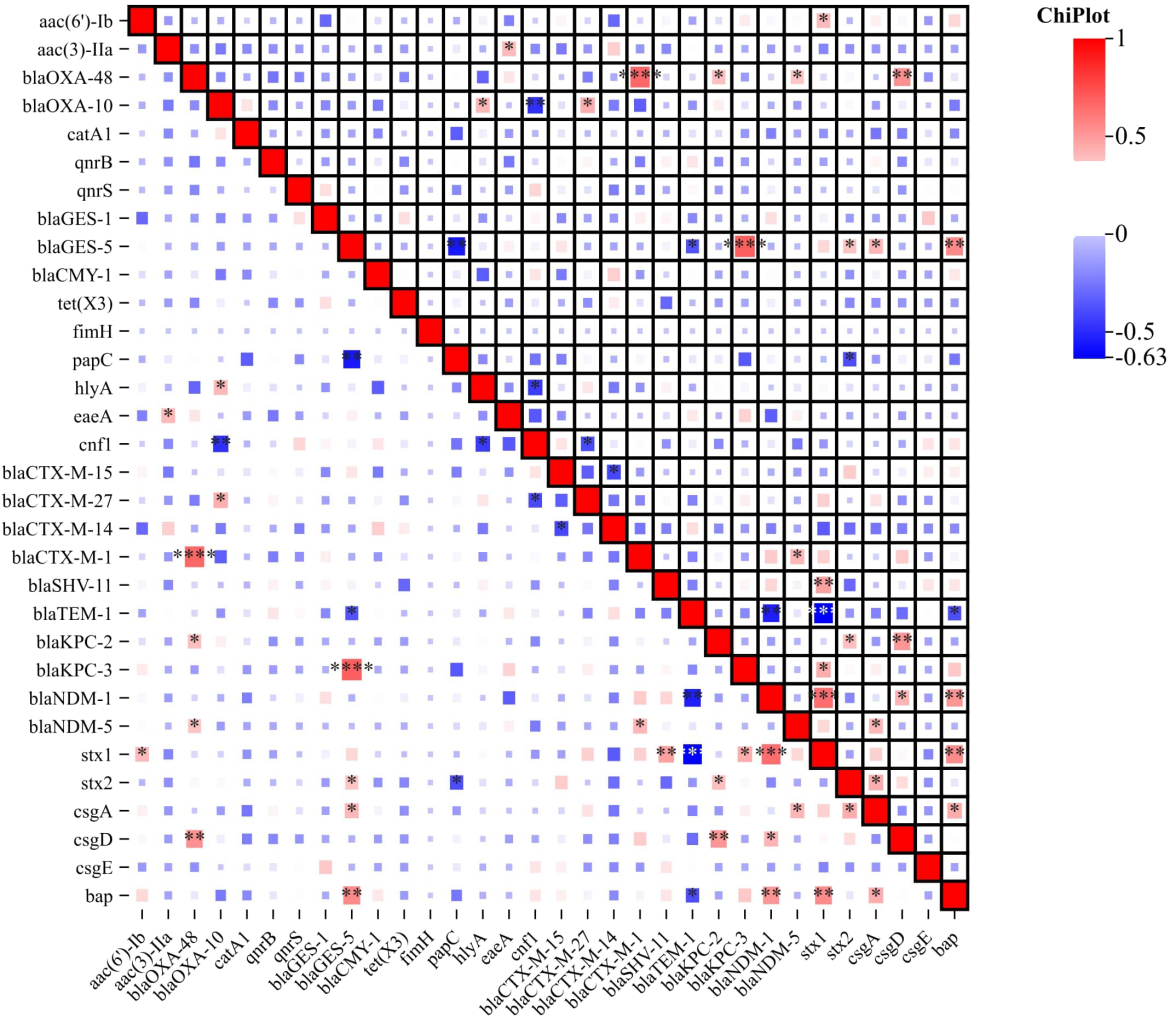
Correlation Heatmap of Resistance, Virulence, and Biofilm-Associated Genes. The heatmap visually represents the correlation between antibiotic resistance genes, virulence genes and biofilm-associated genes among the ESBL-Producing Cephalosporin-Resistant *Escherichia coli* ST410. Color intensity and direction indicate the strength and nature of the correlation, respectively. The correlation heatmap was visualized on the image processing website Chiplot (https://www.chiplot.online, accessed on 15 January 2024). Resistance, Virulence, and Biofilm-Associated Genes -Among Correlative degree low, middle and high. *, low, **, middle, ***, high.

The most commonly detected ESBL genes among the isolates were *bla*_CTX-M_ 89.65% (n=26), indicating the higher presence of extended-spectrum β-lactamase enzymes. The specific types identified were *bla*_CTX-M-15_ 34.48% (n=10), *bla*_CTX-M-14_ 20.69% (n=6), *bla*_CTX-M-27_ 17.24% (n=5), *bla*_CTX-M-1_ 13.79% (n=4), and *bla*_CTX-M-140_ 3.45% (n=1). In addition to *bla*_CTX-M_, other ESBL genes detected included *bla*_TEM_ 79.31% (n=23) and *bla*_SHV_ 62.07% (n=18), which are also associated with β-lactam resistance. The detailed breakdown of ESBL types is as follows: 12 strains with TEM-type ESBLs, 8 strains with SHV-type ESBLs, and 6 strains with CTX-M-type ESBLs. Among the 29 strains, TEM and SHV enzymes were detected; however, these are not classified as ESBLs stricto sensu unless they confer resistance to third-generation cephalosporins, which can be confirmed by a positive double-disc synergy test. Specifically, three strains showed a positive double-disc synergy test but did not harbor any CTX-M enzymes. The detailed breakdown of ESBL types is as follows: 12 strains with TEM-type ESBLs, 8 strains with SHV-type ESBLs, and 6 strains with CTX-M-type ESBLs. Furthermore, the following antibiotic resistance genes were identified in the isolates: *bla*_OXA-1_ 34.48% (n=10), *bla*_OXA-48_ 17.24% (n=5), *bla*_KPC-2_ 10.34% (n=3), *bla*_KPC-3_ 6.89% (n=2), *bla*_NDM-1_13.79% (n=4), *bla*_NDM-5_ 3.45% (n=1), *bla*_GES-1_ 6.89% (n=2), *bla*_GES-5_ 3.45% (n=1), *bla*_CYM-1_ 1.34% (n=3). To provide a comprehensive overview, of the percentage of isolates carrying each resistance gene. In addition, other antibiotic resistance genes were identified as aminoglycoside resistance genes *aac(6’)-Ib* 55.17% (n=16), *aac(3)-IIa* 10.34% (n=3); fluoroquinolone resistance genes *qnrB* 20.69% (n=6), and *qnrS* 13.79% (n=4); chloramphenicol resistance gene *cat*A1 21.14% (n=7), glycylcycline resistance gene *tet*(X3) 13.79% (n=4) (**Figure 4**; **Supplementary Table 3**).

### Virulence and biofilm genomics

Virulence genes were categorized into attachment, biofilm formation, and cellular toxicity/lysis. We analyzed their associations with serotypes and phylogenetic groups, despite the limited sample size. No significant association patterns were observed, suggesting a complex distribution of virulence factors across different serotypes and phylogenetic groups. Among the detected genes, the most prevalent virulence gene was *fimH*, which encodes the type 1 fimbriae adhesin, found in 100% (n=29) of the isolates, indicating its universal presence among the ST410 strains. The *pap*C gene, which encodes P fimbriae facilitating bacterial adherence to host cells, was detected in 82.76% (n=24) of the isolates. The *hly*A gene, associated with hemolysin production, was found in 75.86% (n=22) of the isolates. The *cnf*1 gene, which was identified in 62.07% (n=18) is associated with cytotoxic necrotizing factor production, which can lead to cellular damage while the *eae*A gene was present in 41.38% (n=12) of the isolates. This gene is associated with the formation of attaching and effacing lesions and is commonly found in enteropathogenic *E. coli* strains. The *stx*1 and *stx*2 genes, which encode Shiga toxins, were detected in 27.59% (n=8) and 20.69% (n=6) of the isolates, respectively (**Figure 4**). These toxins are known to be involved in the development of severe gastrointestinal symptoms. In addition to virulence genes, the presence of genes associated with biofilm formation was investigated. The *bap* gene, associated with biofilm-associated protein production, was found in 10.34% (n=3) of the isolates. Genes involved in curli fiber production, including *csg*A, *csg*D, and *csg*E, were detected in 17.24% (n=5), 13.79% (n=4), and 10.34% (n=3) of the isolates, respectively (**Figure 4**). These results highlight the presence of various virulence/toxin genes and biofilm-associated genes in the studied isolates. The detection of these genes suggests the potential pathogenicity of the isolates and their ability to cause severe gastrointestinal symptoms and cellular damage. Furthermore, the presence of biofilm-associated genes indicates the ability of the isolates to form biofilms, which can enhance bacterial survival and contribute to antimicrobial resistance. In addition to characterizing the antibiotic resistance and virulence profiles, we conducted an in-depth analysis of the correlation between resistance genes, virulence genes, and biofilm-associated genes. The correlation heatmap, presented in **Figure 4**, provides a visual representation of the associations between various resistance genes, virulence factors, and biofilm-producing genes. The correlation heatmap elucidates potential relationships between these gene categories, offering valuable insights into the co-occurrence patterns. This comprehensive analysis enhances our understanding of the interconnectedness of resistance, virulence, and biofilm formation in the studied isolates.

### Plasmid and conjugations

**Figure 5:**
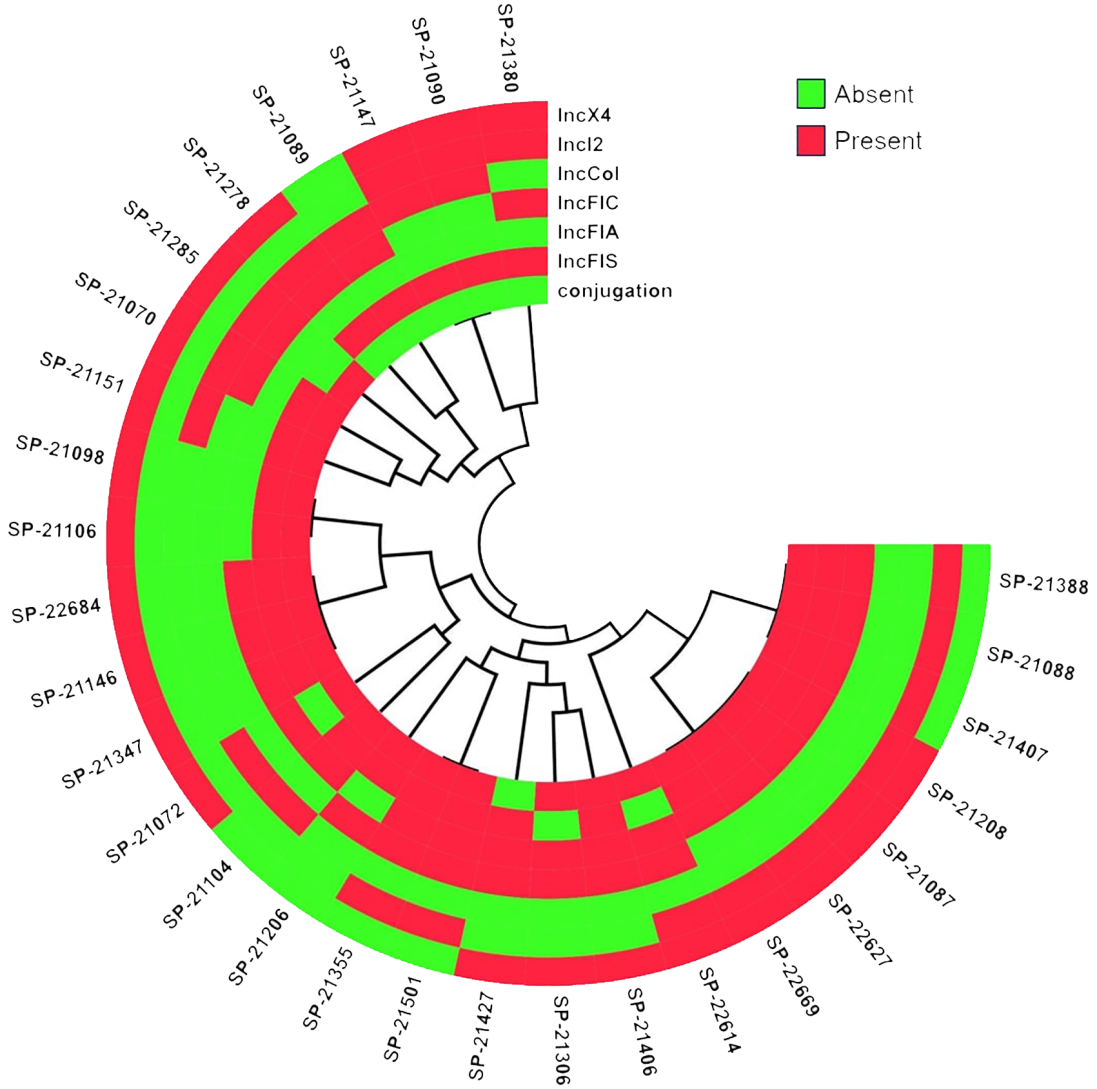
Diversity of Incompatibility type of Plasmids dissemination in ESBL-Producing Cephalosporin-Resistant *Escherichia coli* ST410. The figure illustrates the diverse plasmids identified among ST410 isolates, showcasing a variety of plasmid types. The figure was generated using https://www.chiplot.online/. Last accessed on 15 January 2024.

Diverse plasmids were observed among the ST410 isolates, representing a range of plasmid types. The most commonly identified plasmid IncFIS plasmid, known for its broad host range and association with antibiotic resistance genes, was detected in 93.10% (n=26) of the isolates. The IncX4 plasmid, often associated with the spread of multidrug resistance, was found in 72.41% (n=21) of the isolates. The IncFIA, IncCol, IncI2, and IncFIC plasmids, known to carry various resistance determinants, were identified in 65.51%, 51.72%, 44.82% and 41.37% (n=19), (n=15), (n=13), and (n=12) of the isolates, respectively. These plasmids exhibited varying transmission frequencies, with ranges from 1.3X10^-2^ to 2.7X10^-3^ (**Figure 5**). The presence of multiple plasmid types suggests the potential for horizontal gene transfer and the dissemination of resistance and virulence genes among the ST410 isolates.

## Discussion

The present study focuses on the emergence of a hypervirulent MDR clone of *E. coli* ST410. The genetic characterization of ST410 *E. coli* strains is of utmost importance for several reasons. Firstly, ST410 is considered an international clone that has been associated with various types of infections worldwide (Feng et al., 2019). By studying this clone at the genetic level, we can gain insights into its transmission dynamics, and clinical implications. Understanding the genetic relatedness and evolutionary history of ST410 strains can help elucidate the routes of dissemination and the potential for the spread of antimicrobial resistance and virulence traits. ST410 *E. coli* strains have demonstrated a remarkable ability to acquire and maintain resistance genes, leading to multidrug-resistant phenotypes (Mohamed et al., 2022). Genomic surveillance conducted on carbapenem-resistant *E. coli* (CREC) in Chinese hospitals spanning from 2017 to 2021 uncovered a shift in the predominant sequence type (ST). Notably, ST410 emerged as the most frequently isolated CREC ST during this period, surpassing previously reported prevalent types such as ST167, ST131, and ST617. The prior surveillance studies from 2015 to 2017 had identified ST167, ST131, and ST617 as the most common sequence types, indicating a dynamic shift in the epidemiology of carbapenem-resistant *E. coli* in Chinese hospital settings (Li et al., 2021; Zhang et al., 2017). Investigating the genetic determinants of resistance in these strains can provide crucial information on the mechanisms underlying their resistance profiles. This knowledge is essential for designing effective strategies to combat antimicrobial resistance and preserve the efficacy of antimicrobial agents. Furthermore, our understanding of their virulence potential, emphasizes their pathogenicity and ability to cause severe infections. Additionally, molecular epidemiology aids the development of typing methods and diagnostic tools for rapid identification and surveillance. *E. coli* ST410 strains investigating at the genetic level is crucial for understanding their international clonal nature, antimicrobial resistance patterns, virulence potential, and evolutionary dynamics. Such studies contribute to the broader understanding of *E. coli* pathogenesis and provide valuable information for the development of effective prevention and control strategies. Our study focused on investigating the antimicrobial resistance patterns and genetic characteristics of ST410 *E. coli* isolates. The findings of our research provide valuable insights into the epidemiology and clinical significance of this sequence type. We have established the important study and its contribution to the existing knowledge on ST410 *E. coli*. Phylogenetic group analysis provided that the majority of the isolates belonged to group B2 which is commonly associated with ExPEC strains. Group B2 strains have been frequently implicated in urinary tract infections, sepsis, and other invasive infections while group D is commonly found in the gastrointestinal tract, This observation aligns with previous studies, which report variable distributions of phylogenetic groups across different specimen types (Patil et al., 2019b). Further investigations with larger sample sizes are needed to draw more definitive conclusions. The presence of different phylogenetic groups within the ST410 clone suggests a degree of genetic diversity and highlights the adaptability of this clone to various ecological niches. The presence of diverse serotypes within the ST410 clone suggests the potential for variations in virulence and host tropism among the isolates. Antimicrobial resistance is a global public health concern, and our study revealed significant rates of resistance among the *E. coli* ST410 isolates, particularly against cephalosporins and carbapenems. These findings are consistent with previous studies, demonstrating the persistent and widespread resistance among ST410 *E. coli* strains (Zhong et al., 2022). The high resistance rates observed in our study indicate the urgent need for alternative treatment strategies to combat infections caused by these strains. Among the cephalosporins, a high resistance rate of 100% was observed among the ST410 isolates. Resistance to amoxicillin and carbapenem antibiotics was also prevalent among the isolates. These resistance patterns are of great concern, as cephalosporins and carbapenems are vital antibiotics in clinical practice for the treatment of serious bacterial infections (Vasikasin et al., 2023). The emergence and dissemination of ESBL genes play a significant role in cephalosporin resistance (Mitman et al., 2022). In our study, the most commonly detected ESBL gene was *bla*_CTX-M_, present in 89.65% of the isolates. Among the specific types identified, *bla*_CTX-M-15_ was the predominant variant, detected in 34.48% of the isolates. This finding is consistent with other studies that have reported the dominance of *bla*_CTX-M-15_ in ST410 *E. coli* strains (Hans et al., 2023). The presence of *bla*_TEM_ and *bla*_SHV_ genes, associated with β-lactam resistance, was also notable, with prevalence rates of 79.31% and 62.07% respectively. Carbapenem resistance is a major concern, as these antibiotics are considered last-resort treatments for multidrug-resistant infections. Our study revealed the presence of carbapenem resistance genes, including *bla*_OXA-1_, *bla*_KPC-2_, *bla*_KPC-3_, *bla*_NDM-1_, and *bla*_NDM-5_, among the ST410 isolates. These genes have been associated with various mechanisms of carbapenem resistance, including the production of carbapenemases but enzymes such as CMY-1 hydrolyze carbapenems at very low levels. They are not comparable to carbapenemases like KPC and NDM, enzymes in terms of carbapenem resistance (Li et al., 2021; Sewunet et al., 2022). The detection of these resistance genes underscores the potential dissemination of carbapenem resistance mechanisms in ST410 *E. coli* strains. The high prevalence of the *aac*(6’)-Ib gene suggests a significant resistance to aminoglycosides. While the detection of *qnr*B and *qnr*S genes indicates the potential for fluoroquinolone resistance, these genes were found in only 30% of the strains. This suggests that other resistance mechanisms likely play a more prominent role in conferring resistance to fluoroquinolones in these strains. The observed 0% susceptibility to levofloxacin highlights the complex interplay of multiple resistance mechanisms beyond just the presence of qnr genes. In addition to antimicrobial resistance, our study explored the presence of virulence and biofilm-associated genes in the ST410 isolates. The identification of virulence genes provides insights into the potential pathogenicity of these strains. Among the detected genes, *fim*H, encoding the type 1 fimbriae adhesin, was universally present in all 100% of the isolates (Foroogh et al., 2021), highlighting its crucial role in the colonization and persistence of ST410 *E. coli* strains. The *pap*C gene, associated with P fimbriae-mediated adherence, was detected in 82.76% of the isolates. The *hly*A gene, involved in hemolysin production, was found in 75.86% of the isolates. The *cnf*1 gene, associated with cytotoxic necrotizing factor production, was identified in 62.07% of the isolates. These virulence genes contribute to the pathogenic potential of ST410 *E. coli*, enabling them to cause severe infections and clinical manifestations (Jomehzadeh et al., 2022). Biofilm formation is another important attribute of bacterial pathogenicity and can contribute to antimicrobial resistance (Qian et al., 2022). In our study, we investigated the presence of biofilm-associated genes in the ST410 isolates. The *bap* gene, associated with biofilm-associated protein production, was found in 10.34% of the isolates. Genes involved in curli fiber production, including *csg*A, *csg*D, and *csg*E, were detected. The presence of these biofilm-associated genes suggests the ability of ST410 *E. coli* strains to form biofilms, which can enhance their survival and contribute to persistent infections and antimicrobial resistance (Ogasawara et al., 2011). Comparing our results with recently published data, our study provides valuable insights into the epidemiology and clinical implications of ST410 *E. coli* strains. Our findings align with previous studies, highlighting the persistent antimicrobial resistance and virulence characteristics of ST410 isolates. The high rates of resistance against cephalosporins and carbapenems underscore the need for effective infection control measures and the development of alternative treatment strategies (Mahazu et al., 2021). Studying the transfer dynamics and stability of these plasmids would provide insights into their role in the dissemination of genetic traits within the bacterial population. The results of this study revealed the presence of diverse plasmids among the high-risk clone ST410 isolates. The most commonly identified plasmids, IncFIS and IncX4 are known for their broad host range and association with antibiotic-resistance genes (Patil et al., 2023a; Zhong et al., 2018). This finding suggests that the ST410 isolates have a high potential for carrying and disseminating antibiotic resistance determinants. The varying transmission frequencies of the identified plasmids further highlight their potential for horizontal gene transfer. Horizontal gene transfer plays a crucial role in the spread of antibiotic resistance and virulence determinants among bacterial populations (Sun et al., 2019). The findings of this study are consistent with previous research demonstrating the association between specific plasmids and antibiotic resistance in various bacterial strains (San Millan, 2018). Despite the significant contributions of our study, some limitations should be acknowledged. First, our research was conducted on a limited number of isolates from a specific population, and thus, the generalizability of the findings may be limited. Future studies involving larger sample sizes and diverse populations would enhance the representativeness of the results. Additionally, our study focused primarily on genotypic characterization, and further investigations incorporating phenotypic assays, such as biofilm formation assays and *in vivo* studies, would provide a more comprehensive understanding of the pathogenicity and clinical impact of ST410 *E. coli*.

## Conclusion

Our study sheds light on the antimicrobial resistance, virulence, and genetic characteristics of ST410 *E. coli* isolates. The widespread resistance patterns observed, particularly against cephalosporins and carbapenems, highlight the urgency to implement appropriate infection control measures and develop alternative therapeutic options. The presence of virulence and biofilm-associated genes further emphasizes the potential pathogenicity and persistence of ST410 strains. Our findings contribute to the existing knowledge of ST410 *E. coli* and provide valuable insights for the management and control of infections caused by these strains.

## Data Availability

The datasets presented in this study can be found in online repositories. The names of the repository/repositories and accession number(s) can be found in the article/**Supplementary Material**.

## Supplementary material

Supplementary Table 1 Collected data from the Sequence Read Archive (SRA) at NCBI spanning the years 2009 to 2021.

Supplementary Table 2 Identification of Isolates Identified as *E. coli* based on the API 20E.

Supplementary Table 3 Distribution of resistance genes and virulence genes among 29 ESBLs producing *E. coli.*
