# Supplementary material for: Genetic landscape of ESBL producing international clone ST410 of Escherichia coli from pediatric infections in Shenzhen, China
Source: Front Cell Infect Microbiol. 2024 Sep 11;14:1403234. doi: 10.3389/fcimb.2024.1403234 (PMC11422233; doi:10.3389/fcimb.2024.1403234)
Supplement: Supplementary Table 2 — Identification of Isolates Identified as E. coli based on the API 20E. [file Table2.docx]

**Supplementary file-2**

**Table -1 Identification of Isolates Identified as *E. coli* based on the API 20E**

| **Sr No** | **Isolate ID** | **Test Results** | | | | | | | | | | | | | | | | | | | |
| --- | --- | --- | --- | --- | --- | --- | --- | --- | --- | --- | --- | --- | --- | --- | --- | --- | --- | --- | --- | --- | --- |
|  |  | **ONPG** | **ADH** | **LDC** | **ODC** | **CIT** | **H_2_S** | **URE** | **TDA** | **IND** | **VP** | **GEL** | **GLU** | **MAN** | **INO** | **SOR** | **RHA** | **SAC** | **MEL** | **AMY** | **ARA** |
| 1 | SP-21070 | **+** | **-** | **+** | **+** | **-** | **-** | **-** | **-** | **+** | **-** | **-** | **+** | **+** | **-** | **+** | **+** | **-** | **+** | **-** | **+** |
| 2 | SP-21072 | **+** | **-** | **+** | **+** | **-** | **-** | **-** | **-** | **+** | **-** | **-** | **+** | **+** | **-** | **+** | **+** | **-** | **+** | **-** | **+** |
| 3 | SP-21087 | **+** | **-** | **+** | **+** | **-** | **-** | **-** | **-** | **+** | **-** | **-** | **+** | **+** | **-** | **+** | **+** | **-** | **+** | **-** | **+** |
| 4 | SP-21089 | **+** | **-** | **+** | **+** | **-** | **-** | **-** | **-** | **+** | **-** | **-** | **+** | **+** | **-** | **+** | **+** | **-** | **+** | **-** | **+** |
| 5 | SP-21088 | **+** | **-** | **+** | **+** | **-** | **-** | **-** | **-** | **+** | **-** | **-** | **+** | **+** | **-** | **+** | **+** | **-** | **+** | **-** | **+** |
| 6 | SP-21090 | **+** | **-** | **+** | **+** | **-** | **-** | **-** | **-** | **+** | **-** | **-** | **+** | **+** | **-** | **+** | **+** | **-** | **+** | **-** | **+** |
| 7 | SP-21098 | **+** | **-** | **+** | - | **-** | **-** | **-** | **-** | **+** | **-** | **-** | **+** | **+** | **-** | **+** | **+** | **+** | **+** | **-** | **+** |
| 8 | SP-21104 | **+** | **-** | **+** | **+** | **-** | **-** | **-** | **-** | **+** | **-** | **-** | **+** | **+** | **-** | **+** | **+** | **-** | **+** | **-** | **+** |
| 9 | SP-21106 | **+** | **-** | **+** | **+** | **-** | **-** | **-** | **-** | **+** | **-** | **-** | **+** | **+** | **-** | **+** | **+** | **-** | **+** | **-** | **+** |
| 10 | SP-21147 | **-** | **-** | **+** | **+** | **-** | **-** | **-** | **-** | **+** | **-** | **-** | **+** | **+** | **-** | **+** | **+** | **-** | **+** | **-** | **+** |
| 11 | SP-21146 | **+** | **-** | **+** | **+** | **-** | **-** | **-** | **-** | **+** | **-** | **-** | **+** | **+** | **-** | **+** | **+** | **-** | **+** | **-** | **+** |
| 12 | SP-21151 | **+** | **-** | **+** | **+** | **-** | **-** | **-** | **-** | **+** | **-** | **-** | **+** | **+** | **-** | **+** | **+** | **-** | **+** | **-** | **+** |
| 13 | SP-21206 | **+** | **-** | **+** | **+** | **-** | **-** | **-** | **-** | **+** | **-** | **-** | **+** | **+** | **-** | **+** | **+** | **-** | **+** | **-** | **+** |
| 14 | SP-21208 | **+** | **-** | **+** | **+** | **-** | **-** | **-** | **-** | **+** | **-** | **-** | **+** | **+** | **-** | **+** | **+** | **-** | **+** | **-** | **+** |
| 15 | SP-21278 | **+** | **-** | **+** | **-** | **-** | **-** | **-** | **-** | **+** | **-** | **-** | **+** | **+** | **-** | **+** | **+** | **+** | **+** | **-** | **+** |
| 16 | SP-21285 | **+** | **-** | **+** | **+** | **-** | **-** | **-** | **-** | **+** | **-** | **-** | **+** | **+** | **-** | **+** | **+** | **-** | **+** | **-** | **+** |
| 17 | SP-21306 | **+** | **-** | **+** | **+** | **-** | **-** | **-** | **-** | **+** | **-** | **-** | **+** | **+** | **-** | **+** | **+** | **-** | **+** | **-** | **+** |
| 18 | SP-21347 | **+** | **-** | **+** | **+** | **-** | **-** | **-** | **-** | **+** | **-** | **-** | **+** | **+** | **-** | **+** | **+** | **-** | **+** | **-** | **+** |
| 19 | SP-21355 | **+** | **-** | **+** | **+** | **-** | **-** | **-** | **-** | **+** | **-** | **-** | **+** | **+** | **-** | **+** | **+** | **-** | **+** | **-** | **+** |
| 20 | SP-21380 | **+** | **-** | **+** | **+** | **-** | **-** | **-** | **-** | **+** | **-** | **-** | **+** | **+** | **-** | **+** | **+** | **-** | **+** | **-** | **+** |
| 21 | SP-21388 | **+** | **-** | **+** | **+** | **-** | **-** | **-** | **-** | **+** | **-** | **-** | **+** | **+** | **-** | **+** | **+** | **-** | **+** | **-** | **+** |
| 22 | SP-21406 | **+** | **-** | **+** | **+** | **-** | **-** | **-** | **-** | **+** | **-** | **-** | **+** | **+** | **-** | **+** | **+** | **-** | **+** | **-** | **+** |
| 23 | SP-21407 | **+** | **-** | **+** | **+** | **-** | **-** | **-** | **-** | **+** | **-** | **-** | **+** | **+** | **-** | **+** | **+** | **-** | **+** | **-** | **+** |
| 24 | SP-21427 | **+** | **-** | **+** | **-** | **-** | **-** | **-** | **-** | **+** | **-** | **-** | **+** | **+** | **-** | **+** | **+** | **-** | **+** | **-** | **+** |
| 25 | SP-21501 | **+** | **-** | **+** | **+** | **-** | **-** | **-** | **-** | **+** | **-** | **-** | **+** | **+** | **-** | **+** | **+** | **-** | **+** | **-** | **+** |
| 26 | SP-22614 | **+** | **-** | **+** | **+** | **-** | **-** | **-** | **-** | **+** | **-** | **-** | **+** | **+** | **-** | **+** | **+** | **-** | **+** | **-** | **+** |
| 27 | SP-22627 | **+** | **-** | **+** | **+** | **-** | **-** | **-** | **-** | **+** | **-** | **-** | **+** | **+** | **-** | **+** | **+** | **-** | **+** | **-** | **+** |
| 28 | SP-22669 | **+** | **-** | **+** | **+** | **-** | **-** | **-** | **-** | **+** | **-** | **-** | **+** | **+** | **-** | **+** | **+** | **-** | **+** | **-** | **+** |
| 29 | SP-22684 | **+** | **-** | **+** | **+** | **-** | **-** | **-** | **-** | **+** | **-** | **-** | **+** | **+** | **-** | **+** | **+** | **-** | **+** | **-** | **+** |
| 30 | ATCC29522 | **+** | **-** | **+** | **+** | **-** | **-** | **-** | **-** | **+** | **-** | **-** | **+** | **+** | **-** | **+** | **+** | **-** | **+** | **-** | **+** |

All codes listed identified isolates as *E. coli*. Biochemical tests: ONPG, 2-nitrophenyl-β-D-galactopyranoside to detect β-galactosidase; ADH, arginine dihydrolase; LDC, lysine decarboxylase; ODC, ornithine decarboxylase; CIT, citrate utilization; H2S, H2S production; URE, urease; TDA, tryptophan deaminase; IND, indole production; VP, Voges-Proskauer test for acetoin; GEL, gelatinase; GLU, glucose fermentation; MAN, mannitol fermentation; INO, inositol fermentation; SOR, sorbitol fermentation; RHA, rhamnose fermentation; SAC, saccharose (sucrose) fermentation; MEL, melibiose fermentation; AMY, amygdalin fermentation; ARA, arabinose fermentation; Red shaded areas indicate tests that were different from E. coli ATCC 25922, the control (reference) strain.
