# Supplementary material for: Genetic landscape of ESBL producing international clone ST410 of Escherichia coli from pediatric infections in Shenzhen, China
Source: Front Cell Infect Microbiol. 2024 Sep 11;14:1403234. doi: 10.3389/fcimb.2024.1403234 (PMC11422233; doi:10.3389/fcimb.2024.1403234)
Supplement: Supplementary Table 3 — Distribution of resistance genes and virulence genes among 29 ESBLs producing E. coli. [file Table3.docx]

|  |  |  |  |  | ESBLs | | |
| --- | --- | --- | --- | --- | --- | --- | --- |
| code | Sex | age | Resistance gene | Virulence gene | CTX-M | SHV | TEM |
| SP-21070 | F | 8m | *bla*_OXA-10_,*bla_O_*_XA-48_ | *fim*H,*pap*C,*hly*A ,*eae*A | *bla*_CTX-M-15_ | *bla*_SHV-11_ | *bla*_TEM-1_ |
| SP-21072 | f | 8m | *bla*_CYM-1_,aac(6')-Ib ,*qnr*B ,*qnr*S | *fim*H,*papC*, *hly*A,*cnf*1 | *bla*_CTX-M-27_ | *bla*_SHV-11_ | *bla*_TEM-1_ |
| SP-21087 | f | 8m | *bla*_OXA-10_,aac(6')-Ib ,catA1,tet(X3) | fimH,papC,hlyA, eaeA | *bla*_CTX-M15_ |  | *bla*_TEM-1_ |
| SP-21089 | f | 1 | *aac(6')-Ib* *,qnrB* | fimH,papC,hlyA ,cnf1 | *bla*_CTX-M-15_ | *bla*_SHV-11_ | *bla*_TEM-1_ |
| SP-21088 | m | 1 | *bla*_OXA-10_,qnrS,catA1 | fimH,hlyA ,hlyA ,cnf1 | *bla*_CTX-M-15_ |  | *bla*_TEM-1_ |
| SP-21090 | f | 1 | *bla*_OXA-48,_ *bla*_CYM-1_ | fimH,papC,cnf1,eaeA | *bla*_CTX-M-14_ | *bla*_SHV-11_ | *bla*_TEM-1_ |
| SP-21098 | f | 10 | *qnrB* | fimH,papC,hlyA ,cnf1 | *bla*_CTX-M-15_ | *bla*_SHV-11_ | *bla*_TEM-1_ |
| SP-21104 | m | 10 | *bla*_OXA-10_,aac(6')-Ib | fimH,papC,hlyA ,eaeA | *bla*_CTX-M-27_ | *bla*_SHV-11_ | *bla*_TEM-1_ |
| SP-21106 | m | 9m | *bla*_GES-5_,aac(6')-Ib | fimH,hlyA ,cnf1,eaeA | *bla*_CTX-M-15_ | *bla*_SHV-11_ |  |
| SP-21147 | m | 11 | *bla*_OXA-48_,aac(6')-Ib | fimH,papC,hlyA ,cnf1 | *bla*_CTX-M-1_ |  | *bla*_TEM-1_ |
| SP-21146 | f | 11 | *bla*_OXA-10_,tet(X3) | fimH,papC,hlyA,cnf1 | *bla*_CTX-M14_ |  | *bla*_TEM-1_ |
| SP-21151 | m | 10m | *bla*_GES-1_,tet(X3) | fimH,papC,hlyA,cnf1 | *bla*_CTX-M-1_ | *bla*_SHV-11_ |  |
| SP-21206 | m | 10m | *bla*_OXA-10_,aac(6')-Ib ,catA1 | fimH,papC,hlyA ,cnf1 | *bla*_CTX-M-15_ | *bla*_SHV-11_ | *bla*_TEM-1_ |
| SP-21208 | f | 12 | *bla*_OXA-10_ | fimH,papC,hlyA | *bla*_CTX-M27_ |  |  |
| SP-21278 | f | 13 | *aac(6')-Ib* *,catA1* | fimH,hlyA,cnf1 | *bla*_CTX-M-14_ | *bla*_SHV-11_ | *bla*_TEM-1_ |
| SP-21285 | m | 13 | *aac(6')-Ib* *,aac(3)-IIa* | fimH,papC,hlyA ,eaeA |  | *bla*_SHV-11_ | *bla*_TEM-1_ |
| SP-21306 | m | 13 | *bla*_OXA-48_,aac(6')-Ib | fimH,papC,cnf1 | *bla*_CTX-M-1_ | *bla*_SHV-11_ |  |
| SP-21347 | f | 14 | *bla*_OXA-48_,catA1 | fimH,papC,cnf1,eaeA | *bla*_CTX-M-1_ |  | *bla*_TEM-1_ |
| SP-21355 | f | 15 | *aac(6')-Ib* *,qnrB* | fimH,papC,cnf1 | *bla*_CTX-M15_ |  | *bla*_TEM-1_ |
| SP-21380 | f | 8m | *bla*_OXA-10_,qnrB | fimH,papC,hlyA ,eaeA | *bla*_CTX-M-27_ | *bla*_SHV-11_ | *bla*_TEM-1_ |
| SP-21388 | f | 8m | *aac(3)-IIa* | fimH,papC,cnf1,eaeA | *bla*_CTX-M-14_ |  | *bla*_TEM-1_ |
| SP-21406 | f | 8m | *bla*_OXA-10_,aac(6')-Ib ,catA1 | fimH,papC,hlyA | *bla*_CTX-M-27_ | *bla*_SHV-11_ |  |
| SP-21407 | m | 3 | *bla*_GES-1_,qnrS | fimH,papC,cnf1,eaeA |  | *bla*_SHV-11_ | *bla*_TEM-1_ |
| SP-21427 | f | 3m | *qnrB ,catA1* | fimH,papC,hlyA | *bla*_CTX-M-14_ | *bla*_SHV-11_ | *bla*_TEM-1_ |
| SP-21501 | f | 4 | *bla*_OXA-10_,aac(6')-Ib | fimH,papC,hlyA |  | *bla*_SHV-11_ |  |
| SP-22614 | f | 4 | *aac(3)-IIa* | fimH,papC,hlyA ,eaeA | *bla*_CTX-M-14_ |  | *bla*_TEM-1_ |
| SP-22627 | f | 4m | *bla*_CYM-1_,aac(6')-Ib ,tet(X3) | fimH,papC,cnf1 | *bla*_CTX-M-140_ |  | *bla*_TEM-1_ |
| SP-22669 | m | 5 | *aac(6')-Ib* *,qnrS* | fimH,papC,hlyA ,cnf1 | *bla*_CTX-M-15_ |  | *bla*_TEM-1_ |
| SP-22684 | m | 5m | *bla*_OXA-48_,aac(6')-Ib | fimH,papC, hlyA ,eaeA | *bla*_CTX-M-1_ | *bla*_SHV-11_ | *bla*_TEM-1_ |
